# The Role of Bone Subtraction Computed Tomographic Angiography in Determining Intracranial Aneurysms in Non-Traumatic Subarachnoid Hemorrhage

**DOI:** 10.5812/iranjradiol.12670

**Published:** 2014-05-15

**Authors:** Aysegul Kayhan, Osman Koc, Suat Keskin, Fatih Keskin

**Affiliations:** 1 Department of Radiology, Beysehir State Hospital, Konya, Turkey; 2 Department of Radiology, Meram School of Medicine, Necmettin Erbakan University, Konya, Turkey; 3 Department of Neurosurgery, Meram School of Medicine, Necmettin Erbakan University, Konya, Turkey

**Keywords:** Intracranial Aneurysm, Subtraction Technique, Subarachnoid Hemorrhage, Computed Tomography

## Abstract

**Background:**

The presence of blood in the subarachnoid space is an acute pathology with a serious risk of death and complications. The most common etiology (approximately 80%) is intracranial aneurysm.

**Objectives:**

The aim of this study was to assess the role of bone subtracted computed tomographic angiography (BSCTA), a novel and noninvasive method for determining and characterizing intracranial aneurysms.

**Patients and Methods:**

Sixty consecutive patients with clinically suspected non-traumatic subarachnoid hemorrhage (SAH) were considered to enter the study. The subtraction quality was inadequate in ten patients; thus, they were excluded, leaving 50 patients (84.4%) in the study. Bone subtracted and non-subtracted 3D images were obtained from the BSCTA raw data sets. All images obtained by digital subtraction angiography (DSA), BSCTA, and computed tomographic angiography (CTA) were evaluated for the presence or absence of an aneurysm and the location, minimal sac diameter, and neck size ratio of the aneurysm. DSA was considered as the gold standard during the evaluation of the data.

**Results:**

Of the 50 patients who participated in this study, 11 had no aneurysms as determined by both CTA and DSA. Examination of the remaining 39 patients revealed the presence of 51 aneurysms. While 3D-CTA could not detect six aneurysms that were located in the base of the skull, 3D-BSCTA easily detected them. Moreover, five aneurysms were only partially detected by 3D-CTA. According to this data, the sensitivity of 3D-BSCTA and 3D-CTA was calculated as 98% and 86.3%, respectively; the specificity was calculated as 100% and 90.9%, respectively, per aneurysm; and the sensitivity of 100% for 3D-BSCTA and 98% for 3D-CTA was achieved by using combined images with multi-planar reconstruction (MPR) and maximum intensity projection (MIP). BSCTA detected and characterized the aneurysms as well as DSA, and BSCTA and DSA gave concordant results in detecting aneurysms.

**Conclusions:**

BSCTA is easily accessible, less time consuming, and most importantly, a non-invasive technique for detecting intracranial aneurysms. It is also suitable for patients who have been referred to emergency services. Therefore, it can be used in emergency conditions and as a first-line diagnostic method in patients with non-traumatic SAH.

## 1. Background

The presence of blood in the subarachnoid space is an acute pathology with a serious risk of death and complications. The most common etiology (approximately 80%) is intracranial aneurysm (1). According to studies conducted on autopsy series, the detection of unruptured aneurysms ranges from 1.3 to 7.9% (2). The risks of morbidity and mortality increase day by day in these patients, so it is essential to make an accurate diagnosis and plan the treatment as quickly as possible. Digital subtraction angiography (DSA) has been the gold standard for the detection and characterization of intracranial aneurysms (1, 3, 4). However, even in experienced hands, the most important drawback is an overall risk of complications, ranging from 0.07 to 1% (5-8), as well as the possibility of permanent neurological damage in 5% of patients (6, 7). For this reason, the need to investigate noninvasive methods for detecting intracranial aneurysms has arisen. Computed tomographic angiography (CTA) is the first choice in patients with subarachnoid hemorrhage (SAH) and aneurysms, because it is easily accessible, less time consuming, and most importantly, a noninvasive technique. It is also suitable for patients who have been referred to emergency services (9).

## 2. Objectives

The aim of this study is to assess the role of bone subtracted computed tomographic angiography (BSCTA), a novel, noninvasive method for determining and characterizing intracranial aneurysms.

## 3. Patients and Methods

### 3.1. Study Population

Between June 2009 and May 2010, 60 consecutive patients (29 males, 31 females; age range, 16-84 years; mean age, 50.6 years) with clinically suspected non-traumatic SAH were referred to the emergency department of the our hospital. SAH was diagnosed by the presence of blood in the subarachnoid spaces, using an unenhanced CT scan. The patients were scheduled to undergo multi-detector computed tomographic angiography (MDCTA) and conventional DSA. Exclusion criteria were traumatic SAH, severe clinical status, severe vasospasm and severe vascular calcification. In accordance with these criteria, ten patients (16.6%) with poor- or moderate-quality BSCTAs because of severe vasospasm and non-stabilized movements were excluded. Moreover, patients who did not accept the DSA examination, who had known drug allergies, or serum creatinine levels higher than 1.5 mg/dL were excluded. All patients were informed about the procedures and complications, and approvals were obtained. Relatives signed the approval forms of unconscious patients. Of the 50 patients who were included in this study, subtraction quality was excellent in forty three and good in seven patients. To determine the etiology of SAH, DSAs and BSCTAs were performed within the first 24-72 hours in all patients. BSCTA was conducted before DSA when possible, and to prevent contrast overload, a gap of 12-24 hours was allowed between the two tests.

### 3.2. Scanning Protocol

BSCTAs were performed with a 64-detector device (Somatom Sensation 64; Siemens Medical Systems, Forchheim, Germany). After obtaining the lateral scanogram, scanning was conducted in the craniocaudal direction from the level of the C2 vertebra through the vertex. The acquisition and reconstruction parameters were defined as follows: 0.6 mm collimation, 1.2 mm pitch, scan time of 4.07 seconds, 100 kV, 160 mAs, FOV 212 mm. Immobilization was achieved by using adhesive bands on unconscious patients. Special software was used for the subtraction process. The mean processing time of the images was 4.1 minutes. A low-dose, non-enhanced scan was obtained first, and in the few seconds immediately following, an enhanced CTA was performed. To conduct the enhanced CTA, 100 mL non-ionic contrast material (Visipaque 320 mg; Amersham Health, Cork, Ireland) was administered through the antecubital vein with an automatic injector (Ulrich, Medizin Technik, Ulm, Germany) at a flow rate of 4 mL/s. At 5 seconds after the initiation of administering the contrast material, repetitive low-dose monitoring scans in the skull base were obtained using axial images (120kV, 20mAs). The scan was initiated when the contrast material was seen in the proximal segment of the internal carotid artery (ICA) (semi-automatic triggering). No complications were detected in the 50 patients participating in this study. All the images were reconstructed with 0.75 section thickness and at increments of 0.75.

### 3.3. Data Analysis

All the data sets were analyzed on a workstation (Syngo MMWP; Siemens Medical Solutions, Forchheim, Germany) with a special software (Neuro DSA-CT, VE 22 A; Siemens Medical Solutions). BSCTA images were automatically generated by subtracting the non-enhanced data from the enhanced data using the software, and a subtracted new data set in digital imaging and communications in medicine (DICOM) format was obtained. The data set emphasized the soft tissues and vascular structures, especially those located in the skull base, which therefore appeared more prominent. During the subtraction process, bone remnants were observed in the unstabilized patients due to an insufficient overlap of bone structures. When the patients’ movements were less than 30° in a single direction, the angle differences were corrected on the workstation before the subtraction process. In spite of the various post-processing methods employed, adequate quality was not achieved in two patients. The subtracted data sets were examined using multi planar reconstruction (MPR) and volume rendering technique (VRT) modalities at different angles, opacity values and planes. The conventional CTA images that were obtained from the same examination were examined using MPR, maximum intensity projection (MIP), and VRT images in which user-dependent bone removal was used. BSCTA-VRT images were reviewed for image quality based on the parameters of quality of subtraction, visibility of the intracavernous part of the ICA, and visibility of the ophthalmic arteries (4, 5). According to these criteria, the quality of the images was identified as excellent, good, and moderate:

-Excellent quality: The vascular structures were visible without any artifacts or residual bone; the ophthalmic artery was visible from the origin of the ICA to the intraorbital region; and the cavernous sinus was not visible at all.
-Good quality: The vascular structures were visible, with some bone remnants that did not affect a free view of the vascular structures; and the visible part of the cavernous sinus did not impinge on the evaluation of the intracavernous part of the ICA.
-Moderate quality: The vascular structures were visible, with large bone remnants that affected a free view of the vascular structures; the ophthalmic artery was visible at least at its origin; and the cavernous sinus was visible, although it affected a direct view of the ICA.

Catheter angiography was obtained with a DSA single plane device (GE Advantx LCA, USA), using the Seldinger technique and local anesthesia through a percutaneous femoral catheter. The acquisition and reconstruction parameters were defined as follows: 80 kV, 400 mAs, 1012×1012 matrix and 32 cm FOV. A pigtail catheter was used during the examination. A total of 100-150 mL non-ionic contrast material (Ultravist 300/100 mg iodine/mL; Schering, Berlin, Germany) was administered with an automatic injector (Mark V ProVis; Medrad, Indianola). When sufficient contrast reflux was obtained, only a three-vessel angiography was performed; otherwise, a four-vessel angiography was performed. Towne, lateral, and anteroposterior projections were obtained routinely, and if necessary, additional projections were included. No complications were observed.

All images obtained by DSA, CTA, and BSCTA were evaluated for the presence or absence of aneurysms, as well as the location, minimal sac diameter and neck size ratio of the aneurysm. In the literature published to date, a threshold value of 3 mm has been defined for the size of the CTA; we used this value as the threshold value in this study as well. Accordingly, we classified all the aneurysms into four groups, as follows: smaller than 3 mm, 3-5 mm, 5-15 mm, and 15-25 mm. In addition, aneurysms were classified as saccular or fusiform, according to Yasargil’s classification. The neck of the aneurysm was defined as “wide” if the ratio of the aneurysm transverse diameter to the aneurysm neck diameter was less than 2, and “narrow” if this ratio was more than 2. Aneurysm locations were classified statistically in three groups, as follows: Group 1, located in the anterior cerebral artery (ACA) and anterior communicating artery (ACoA); Group 2, located in the ICA; and Group 3, located elsewhere. The percentages were calculated based on the localization of intracranial aneurysms.Finally, all the data obtained from the DSA, BSCTA, and conventional CTA were compared; the DSA images were used as reference standards.

### 3.4. Statistical Analysis

Standard statistical software (SPSS 16; SPSS Inc., Chicago, IL) was used for statistical testing. Sensitivity, specificity, positive predictive value, and negative predictive value were calculated on per patient and per aneurysm bases for all examination modalities. Sensitivity and specificity of BSCTA and CTA were calculated, and the results were evaluated by Wilcoxon two-sample tests.

## 4. Results

Of the 50 patients who participated in this study, 11 had no aneurysms as determined by both CTA and DSA. Perimesencephalic SAH was detected in eight patients, and parenchymal hemorrhage was detected in three. Because their clinical statuses showed improvement, no additional tests were carried out on these patients. During the initial DSA and BSCTA, two patients experienced severe vasospasms, and no aneurysms were detected. However, the evaluation of the vascular structures was thought to be inadequate due to the presence of vasospasm, and after 15 days these two patients had second DSAs and BSCTAs. According to the second DSA examinations, a right ACA-A1 4.2×4 mm aneurysm was detected in one patient, and a right superior cerebellar artery (SCA) origin 3.9×2.7 mm aneurysm was detected in the other. While the aneurysm that was located in the ACA-A1 location could be seen perfectly in the second BSCTA examination, the aneurysm in the case that originated in the SCA could not be detected with BSCTA-VRT or conventional CTA-VRT images. This could be due to the small size of the aneurysm, its location, and the ongoing vasospasm. Examination of the remaining 39 patients revealed the presence of 51 aneurysms. Four patients had two aneurysms, two patients had three aneurysms, and one patient had five aneurysms.

**Figure 1.**
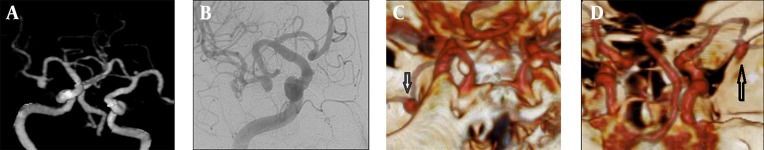
A 53-year-old woman with right MCA bifurcation aneurysm A) BSCTA anteroposterior view; B) DSA anteroposterior view; C) CTA superoinferior view; D) CTA posteroanterior view; the aneurysm in bifurcation of the right MCA was partially detected in CTA (arrows).

**Figure 2.**
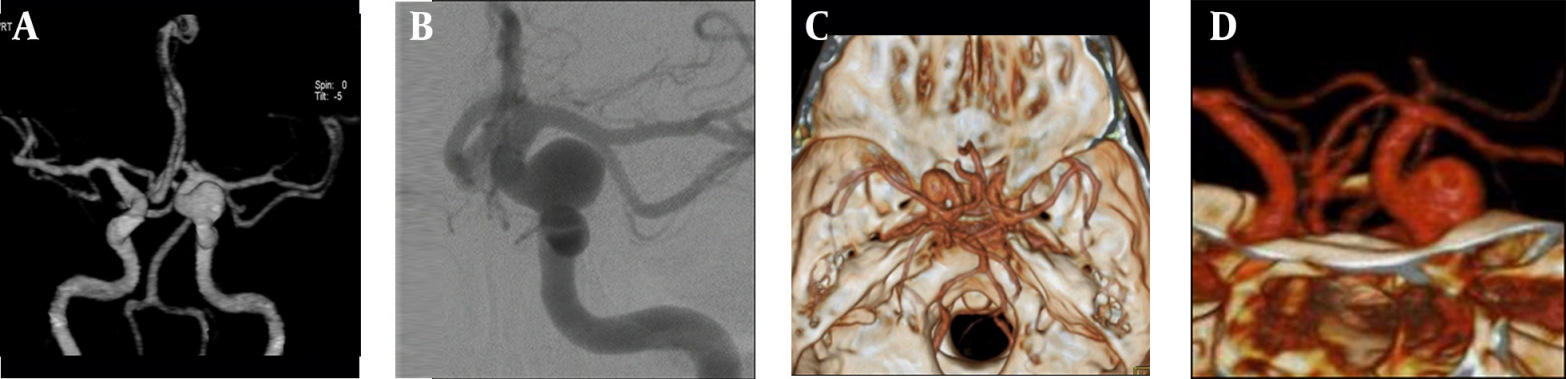
A 55-year-old woman with a left ICA aneurysm A) BSCTA anteroposterior view; B) DSA lateral view; C) CTA superoinferior view; D) CTA lateral view. The aneurysm located in the left ICA was partially detected in CTA, and very clearly in BSCTA.

Of the seven patients with multiple aneurysms, three were male and four were female. A total of seven aneurysms were detected in the three male patients; two of them had two aneurysms and one had three aneurysms. A total of 12 aneurysms were detected in the four female patients; two of them had two aneurysms, one had a three aneurysms, and one had five aneurysms (Figures 1 A-D and 2 A-D).

The percentages of the localization of intracranial aneurysms are shown in Table 1. Sensitivity (94.4% for BSCTA and 74% for CTA) was also calculated for 17 aneurysms localized in the ICA. The relationship between aneurysm size and detection ratio is shown in terms of sensitivity in Table 2 for all aneurysms. When the ratio of the aneurysm transverse diameter to the aneurysm neck diameter was compared, 24 aneurysms with narrow necks and 25 aneurysms with wide necks were determined.

**Table 1.** Anatomical Distribution of Aneurysms^a^

	No.	Percent
**Internal Carotid Artery**	17	33.3
**Anterior Cerebral Artery/ACoA **	15	29.4
**Middle Cerebral Artery**	13	25.4
**Vertebral Artery**	2	3.9
**Basilar Artery**	3	5.8
**Posterior Cerebral Artery and Others **	1	2

^a^ Abbreviation: ACoA, anterior communicating artery

**Table 2.** Relationship Between Aneurysm Size and Detection Ratio

	BSCTA			CTA		
	< 3 mm	> 3 mm	Total	< 3 mm	> 3 mm	Total
**Detected Aneurysm, No.**	13	37	50	8	36	44
**Undetected Aneurysm, No.**	1	0	1	6	1	7
**Sensitivity, %**	93	100	98	57	97	86

One false negative and fifty true positive results were obtained in our series with BSCTA-VRT. The quality of subtraction was evaluated as good (not excellent) in the false negative patient. In one false negative patient, the thin vascular structures could not be adequately determined due to vasospasm and the size of the aneurysms was smaller than 2 mm. Forty-four true positive and seven false negative results were obtained in our series with CTA-VRT. Four false negative aneurysms were located in ICA segments that were close to the skull base and two were smaller than 2 mm. One aneurysm was detected only in DSA, but not in BSCTA or CTA-VRT images. Two aneurysms were missed in the first assessment of BSCTA, but they were identifiable on the review of the respective BSCTA and CTA images. The smallest aneurysm was undetectable in both examinations, despite the re-evaluation.

Finally, the diagnostic accuracy of 3D-BSCTA and 3D-CTA were calculated per patient and per aneurysm by using DSA as the gold standard (Table 3). Six aneurysms in two patients were identified by using combined method with MIP and MPR. Overall diagnostic accuracy was calculated on per patient and per aneurysm bases using 3D images combined with MPR and MIP images (Table 4).

The sensitivity of 3D-BSCTA and 3D-CTA was calculated as 98% and 86.3 respectively, per aneurysm. Sensitivity using combined images with MPR and MIP were calculated as 100% and 97.4%, respectively.

**Table 3.** Diagnostic Indices of 3D-BSCTA and 3D-CTA Calculated per Aneurysm and per Patient^a^

	3D-BSCTA		3D-CTA	
	All Aneurysms	All Patients	All Aneurysms	All Patients
**Sensitivity (%)**	98	97.4	86.3	92.3
**Specificity (%)**	100	100	90.9	90.9
**Positive Predictive Value (%)**	100	100	97.8	97.3
**Negative Predictive Value (%)**	91.7	91.6	58.8	76.9

^a^Abbreviations: 3D-BSCTA, three-dimensional bone subtracted computed tomographic angiography; 3D-CTA: three-dimensional computed tomographic angiography

**Table 4.** Diagnostic Indices Calculated per Aneurysm and per Patient Using 3D Images Combined with MPR and MIP Images ^a^

	3D-BSCTA		3D-CTA	
	All Aneurysms	All Patients	All Patients	All Aneurysms
**Sensitivity (%)**	100	100	97.4	98
**Specificity (%)**	100	100	90.9	90.9
**Positive Predictive Value (%)**	100	100	97.4	98
**Negative Predictive Value (%)**	100	100	90.9	90.9

^a^Abbreviations: 3D-BSCTA, three-dimensional bone subtracted computed tomographic angiography; 3D-CTA: three-dimensional computed tomographic angiography

While a statistically significant difference was observed between 3D-CTA and DSA (z = -2.646, P = 0.008; Wilcoxon two-sample test), there was no statistically significant difference in aneurysm detection between 3D-BSCTA and DSA (z = -1, P = 0.317). In addition, a statistically significant difference was observed between the 3D-CTA and combined CTA (VRT, MPR, MIP) images (z = -2.449, P = 0.014). The sensitivity of BSCTA was found to be higher than that of CTA in aneurysm detection (Table 2).

## 5. Discussion

It is estimated that the annual rate of aneurysm rupture is 0.5-2% (10), and the bleeding risk increases with the increase in diameter (11, 12). Thus, in these patients, making an accurate diagnosis as quickly as possible is life-saving. In recent years, although DSA is still accepted as the gold standard, CTA has become increasingly important in the diagnosis of aneurysms (4, 13, 14). The diagnostic performance of CTA in patients with non-traumatic SAH has improved significantly with the introduction of multi-detector technology (15), thereby increasing the detection rates of intracranial aneurysms (8, 16). In spite of these technological developments, the insufficiency of conventional CTA to image vascular structures close to the bone in the base of the skull has been highlighted (5, 17, 18). Many methods have been developed to overcome this problem; one method is bone subtraction, whereby bone structures in the skull base are subtracted and an image consisting of only vascular structures is obtained.

 In the present study, we examined the diagnostic accuracy of a new radiological method called BSCTA that subtracts signals from the bone automatically. Different methods have been proposed for eliminating bone from CTA data sets in recent studies, but most were time consuming and user-dependent, and thus, not applicable for clinical work. The idea of subtracting non-enhanced data from enhanced CT data was first published by Gorzer(18) in 1994. The closest method to the bone subtraction technique reported in the current study was used by Venema et al. (19). The most important drawback of that study was that the image processing time was too long (approximately one hour); in recent studies, this time has been decreased to 15 minutes (19, 20). In the current study, we were able to decrease this image processing time to only 1 minute using BSCTA. In addition, our method did not require any special user interaction.

In a review of the recent studies regarding the diagnostic accuracy of CTA in detecting intracranial aneurysms, Sakamoto et al. (21) reported a series of 29 carotid cave aneurysms that were all detected by 3D-BSCTA as DSA. The authors, therefore, emphasized that 3D-BSCTA could be an alternative method to DSA when evaluating ICA aneurysms located in the base of the skull. In other studies, Imakita et al. (22) and Abrahams et al. (23) reported, in two series consisting of 49 and eight aneurysms, respectively, that the diagnostic accuracy of 3D-BSCTA was as good as that of DSA. Recently, Romijn et al. (14) investigated the diagnostic performance of subtracted CTA by using the matched mask bone elimination technique and reported a sensitivity of 99% for aneurysms larger than 3 mm. They suggested that DSA was necessary only for patients whose CTA quality was inadequate for evaluating aneurysms.

Tomandl et al. (17) reported that four intracavernous and three paraclinoid aneurysms that were not detected with CTA were obviously seen with BSCTA; Sakamoto et al. (24) reported the same results in one cavernous ICA aneurysm. More recently, Li et al. (4) demonstrated that both CTA and BSCTA detected aneurysms that were located in ACA, MCA, and ACoA as well as DSA did, but the diagnostic performance of CTA was significantly diminished when the aneurysm was located in the ICA. In the current study, the numbers of ACA and ACoA aneurysms were less, but the numbers of ICA aneurysms were more than the other studies. Due to the more localized ICA aneurysms that were located in the skull base, our samples were more appropriate for examining the effectiveness of BSCTA and assessing the superiority of BSCTA to CTA.

It is of interest to understand whether BSCTA can detect aneurysms smaller than 3 mm. In 1994, Schwartz et al. (25) reported that they could not detect aneurysms smaller than 3 mm using CTA. More recently, Teksam et al. (8) reported a 100% sensitivity of CTA for the detection of aneurysms larger than 4 mm, while the sensitivity decreased to 92.6% for aneurysms smaller than 4 mm. The high ratios reported in that study could be due to the consideration of a threshold value of 4 mm (not 3 mm) and the use of a combined evaluation method. In our study, when we used a threshold value of 3 mm for 3D-BSCTA, the sensitivity was 93% for aneurysms smaller than 3 mm and 100% for aneurysms larger than 3 mm. When we used the same threshold value for CTA, the sensitivity was 57.3% for aneurysms smaller than 3 mm and 97% for aneurysms larger than 3 mm.

Evaluation of VRT images from many different angles is possible with 3D-BSCTA, but not with 2D-DSA. In our study, as reported previously by Li et al. (4), we noticed that the vascular relations could be demonstrated more clearly by using this property. We demonstrated that the full neck of the aneurysm and any artery originating from inside the aneurysm could be seen better and more easily by examining the image from different angles (21, 24, 26).

Morhard et al. (27) reported that examining 3D-BSCTA images was easier and less time consuming than examining 3D-CTA images. These authors calculated the average examination time for 3D-CTA and 3D-BSCTA and reported a reduction in examination time from 4.60 minutes to 3.49 minutes. In the current study, we calculated the average duration as 4.10 minutes for both subtraction and examination. Villablanca et al. (6) compared the efficacy of 2D and 3D images in detecting intracranial aneurysms and reported that 10% of intracranial aneurysms may not be identified using only 3D images. Similar to that report, many other reports in the literature have emphasized that using combined methods (VRT, MPR and MIP) always provided more effective results (24). In the present study, we calculated the diagnostic performance of BSCTA and CTA by using only 3D and combined images.

As a different method compared with DSA, Zhang et al. (28) reported that automatic bone removal dual-energy CTA exhibited diagnostic accuracy in evaluating aneurysms; there was no statistical difference between dual-energy CTA and CTA. The first limitation of our study was the increased radiation dose. Despite the additional non-enhanced base CTA dose, the total radiation dose using BSCTA was lower than the DSA dose. The other limitation was that a comparison was carried out between BSCTA and 2D-DSA, and not 3D-DSA. 3D-DSA is a more efficient method, but it is also more expensive and generally difficult to establish in most centers (4, 8, 16, 29). In addition, the spatial resolution of all CTA methods is lower than that of DSA. Thus, very thin arteries (< 1 mm), such as the thalamoperforating and anterior choroidal arteries, are difficult to detect by CTA. Moreover, collateral circulation could not be demonstrated as effectively by CTA as it could by DSA (30).

In conclusion, compared to CTA, BSCTA is a non-invasive method that can detect intracranial aneurysms with high sensitivity at any location, even very close to the base of the skull. BSCTA can detect intracranial aneurysms with high sensitivity that seems to be equivalent to 2D-DSA. Taking the advantages of BSCTA into account, this method appears to be easy and rapid and does not require any user interaction. Thus, it can be used in emergency conditions and as a first-line diagnostic method in patients with non-traumatic SAH.
